# Emotional prosody modulates attention in schizophrenia patients with hallucinations

**DOI:** 10.3389/fnhum.2013.00059

**Published:** 2013-03-04

**Authors:** L. Alba-Ferrara, G. A. de Erausquin, M. Hirnstein, S. Weis, M. Hausmann

**Affiliations:** ^1^Department of Psychiatry and Neurosciences, Roskamp Laboratory of Brain Development, Modulation and Repair, Morsani College of Medicine, University of South FloridaTampa, FL, USA; ^2^Department of Psychology, Durham UniversityDurham, UK; ^3^Department of Biological and Medical Psychology, University of BergenBergen, Norway

**Keywords:** top-down, bottom-up, emotion, attention, implicit prosody, lateralization, schizophrenia, hallucination

## Abstract

Recent findings have demonstrated that emotional prosody (EP) attracts attention involuntarily (Grandjean et al., 2008). The automat shift of attention toward emotionally salient stimuli can be overcome by attentional control (Hahn et al., 2010). Attentional control is impaired in schizophrenia, especially in schizophrenic patients with hallucinations because the “voices” capture attention increasing the processing load and competing for top-down resources. The present study investigates how involuntary attention is driven by implicit EP in schizophrenia with auditory verbal hallucinations (AVH) and without (NAVH). Fifteen AVH patients, 12 NAVH patients and 16 healthy controls (HC) completed a dual-task dichotic listening paradigm, in which an emotional vocal outburst was paired with a neutral vocalization spoken in male and female voices. Participants were asked to report the speaker's gender while attending to either the left or right ear. NAVH patients and HC revealed shorter response times for stimuli presented to the attended left ear than the attended right ear. This laterality effect was not present in AVH patients. In addition, NAVH patients and HC showed faster responses when the EP stimulus was presented to the unattended ear, probably because of less interference between the attention-controlled gender voice identification task and involuntary EP processing. AVH patients did not benefit from presenting emotional stimuli to the unattended ear. The findings suggest that similar to HC, NAVH patients show a right hemispheric bias for EP processing. AVH patients seem to be less lateralized for EP and therefore might be more susceptible to interfering involuntary EP processing; regardless which ear/hemisphere receives the bottom up input.

## Introduction

Behavioral studies suggest that schizophrenia patients show deficits in explicit judgments of emotional prosody (EP) (Rossell and Boundy, 2005; Bozikas et al., 2006; Hoekert et al., 2007; Bach et al., 2009). EP is a non-verbal component of language which allows inferring feelings expressed in speech by encoding/decoding variations in pitch amplitude and tempo. EP decoding is usually assessed *explicitly* that is, participants explicitly attend to stimuli they are asked to classify (Buchanan et al., 2000; Kotz et al., 2003). Since in most everyday situations humans are not specifically asked to focus on EP, implicit prosody paradigms (Sander et al., 2005; Aue et al., 2011) are closer to real life settings.

Implicit emotional processing occurs because emotionally enhanced stimuli capture attention regardless of conscious or voluntary engagement (Bradley and Lang, 2000; Vuilleumier, 2005; Lipp and Waters, 2007). Attraction of attention toward certain objects is a typical bottom-up process, involuntary, automatic, and driven by external stimulus (Pessoa et al., 2002). Alternatively, voluntary attention refers to the top-down, effortful direction of attention under control of the individual, such as when participants are instructed to make an explicit judgment of the emotional tone conveyed in prosodic stimuli (Pattyn et al., 2008).

A few prosody studies manipulated involuntary and voluntary attention, to investigate the influence of emotional salience over top-down control (Grandjean et al., 2005; Sander et al., 2005; Aue et al., 2011), assessing implicit processing of EP while participants listened to dichotically presented male or female voices in either angry or neutral tone. Participants were asked to attend to either the left or right ear, and to make judgments about the speaker's sex of the attended ear disregarding the voice emotional tone. The results revealed that angry prosody attracts attention, provoking behavioral and physiological changes (e.g., skin conductance variations) even when not voluntarily attended to (Aue et al., 2011). Moreover, angry (relative to neutral) stimuli, resulted in increased activation in right temporal areas regardless of stimulus laterality and attentional status, suggesting again that EP is a prepotent stimulus (Grandjean et al., 2005). Reaction times (RTs)in the same task show that when the emotional stimuli converge on the side of the attended ear, such a distractor is more difficult to ignore (Sander et al., 2005); perhaps suggesting dominance of the left ear/right hemisphere for processing auditory emotional stimuli (Grimshaw et al., 2003), such that even if prosody is task-irrelevant, the dominant hemisphere for the task will automatically process the emotional input. Right hemisphere dominance has been repeatedly reported for EP processing in healthy controls (HC) (Ross et al., 1997; Wildgruber et al., 2005).

Implicit perception of EP in schizophrenia has been assessed scantly. A recent study compared schizophrenia patients with controls in two conditions. In the explicit prosody condition, participants attended to semantically neutral words spoken in emotional tones of voice and judged the emotion conveyed by the tone of voice. In the implicit prosody condition participants listened to words with emotional meanings spoken in different prosodic tones and were asked to judge the semantic content of the word ignoring the tone of voice. Importantly, prosodic tone and semantic meaning were either congruent or incongruent (Roux et al., 2010). Schizophrenia patients revealed higher error rates during implicit prosody processing in incongruent trials than HC did, but were not different in response times (which were slower for incongruent trials in both groups). These results suggest that schizophrenia patients are still sensitive to implicit prosody processing (Roux et al., 2010). The increase in error rates may indicate a lack of top-down control (voluntary attention) in the presence of bottom-up salience due to the prosodic features of the stimuli which by capturing attention interfere with the semantic task.

Some studies have found that deficits in EP in schizophrenia patients are linked to basic auditory deficit abnormalities (Leitman et al., 2005, 2011; Jahshan et al., 2012), neurocognitive deficits (Kee et al., 1998), or negative symptoms (Hoekert et al., 2007); whether other did not find an association between emotional perception and negative symptoms (Kee et al., 2003). Instead, Kee and colleagues proposed that emotion processing may be a key mediator between basic neurocognitive abilities and functional outcome (Kee et al., 2003). Most notably to the hypothesis tested in this manuscript, difficulties in EP perception in schizophrenia appear to be strongly associated with the presence and/or history of auditory hallucinations (Rossell and Boundy, 2005; Shea et al., 2007; Kang et al., 2009). Yet, these studies investigated explicit (instead of implicit) EP, limiting their ecological validity. In any case, it has been proposed that the capture of involuntary attention by negative emotional stimuli provides a mechanism for the formation of positive symptoms that is a negative affective bias disturbs contextual processing loosening association of ideas leading to delusions or impeding discrimination between relevant and irrelevant stimuli (Mohanty et al., 2008). A review article on the formation of hallucinations suggests that in auditory verbal hallucination (AVH) patients attention is involuntarily oriented toward certain features of prosody, particularly negative tones, congruent with the emotional valence of most of the AVH that patients suffer from (Alba-Ferrara et al., 2012). If a patient cannot ignore an emotional stimulus even if it is irrelevant or outside the focus of selective attention, such emotional stimulus may gain access to processing in detriment of more relevant external stimuli triggering abnormal perceptions (Alba-Ferrara et al., 2012). There is only one study investigating implicit EP in schizophrenia patients (Roux et al., 2010), which did not, however, differentiate between patients with prominent positive and negative symptoms. The present study therefore investigates implicit EP processing in schizophrenia comparing hallucinators (AVH) with non-hallucinators (NAVH).

The brain representation of EP in normal populations as well as in schizophrenia patients is still a matter of debate. Numerous neuroimaging studies show that emotional prosodic perception involves the interplay between right and left auditory cortical as well as frontal regions (Kotz et al., 2006; Wildgruber et al., 2006; Alba-Ferrara et al., 2011). The right hemisphere is currently thought to predominantly process low frequency modulation signals such as prosody, as well as more articulated tonotopic maps, whereas the left hemisphere has better precision for very fast auditory changes, timing, and lexical properties of speech (Zatorre et al., 1992; Liegeois-Chauvel et al., 2001; Zatorre, 2001; Zatorre and Belin, 2001). However, it should be noted that in a healthy population activation in the left auditory region decreases significantly when the verbal complexity of the prosodic stimulus is reduced, showing that once extract phonetic-segmental information is eliminated, the left hemisphere is not necessary for the prosodic task (Mitchell and Ross, 2008).

Difficulties in implicit EP in hallucinating patients may result from aberrant brain organization. Specifically, AVH patients may display an atypical lateralization of EP, resulting in its impaired processing (Mitchell et al., 2004). EP lateralization in schizophrenia has been extensively debated in the literature (Mitchell et al., 2004; Mitchell and Crow, 2005; Bach et al., 2009), but results remain controversial. Mitchell and colleagues (2004) claim that lateralization is reversed in AVH schizophrenia patients who displayed greater involvement of the left temporal lobe in prosody processing, whereas Bach and colleagues (Bach et al., 2009) found enhanced right lateralization to prosody in these patients. Further clarification is urgently needed.

To investigate the effects that EP exerts on involuntary attention we adopted a dichotic listening paradigm which allows us to investigate EP lateralization (Grandjean et al., 2005; Sander et al., 2005; Aue et al., 2011), to determine whether emotional salience can modulate the allocation of attention. We hypothesized that participants perform generally lower in a gender voice recognition task when prosody stimuli are spoken with a happy or angry voice, especially when emotional stimuli are presented to the attended ear, because both stimuli (i.e., gender prosody and EP) would be processed in the same hemisphere and are thus more likely to interfere. Second, we predict that gender voice recognition in AVH patients is especially susceptible to EP interference compared to NAVH patients and HC. Given that EP lateralization is assumed to be abnormal in schizophrenia, and particularly related to hallucinations, we hypothesized that contrary to the NAVH and HC groups, AVH patients would be distracted by EP even when attending toward the ear opposite to the emotional stimuli.

## Methods

### Participants

Twenty seven (21 males) individuals who met the DSM-IV criteria (American Psychiatric Association, 2000) for schizophrenia were recruited from several outpatient clinics from Northumberland, Tyne and Wear NHS foundation trust and Tees, Esk and Wear Valleys NHS Foundation Trust. The psychiatric diagnosis was confirmed by an independent psychiatrist. All patients were taking antipsychotic drugs (see Table 1). An experienced psychiatrist examined the patients to ensure they did not meet any of the following exclusion criteria: current presence and/or history of co-morbidities with axis I disorders of the DSM or existence of neurological condition including head trauma and long periods of loss of consciousness. Additionally, 16 healthy participants (11 males) were recruited via advertisement in the local post office. They were also screened for history of psychiatric and neurological illness and drug use using the Schedules for Clinical Diagnosis in Neuropsychiatry (SCAN, WHO) to exclude psychopathology. Exclusion criteria for controls were the presence (current or history) of any axis I and II disorders of the DSM.

**Table 1:** **Number of participants for medication type**.

**Drug**	**AVH**	**NAVH**
Haloperidol	2	4
Aripiprazole	3	1
Clozapine	3	2
Olanzapine	1	2
Risperidone	4	3

Number of patient taking typical and atypical antipsychotic medication between groups: schizophrenia patients with hallucinations (AVH) and schizophrenia patients without hallucinations (non-AVH).

### Psychopathology assessment

Additional interviews in patients were conducted by a qualified clinical psychologist, using the Comprehensive Assessment of Symptoms and History (CASH) (Andreasen et al., 1992). This semi-structured interview includes the Scale for the Assessment of Positive Symptoms (SAPS) (Andreasen, 1984) and the Scale for the Assessment of Negative Symptoms (SANS) (Andreasen, 1983). Both diagnostic scales require participants to report symptoms during the week prior to the assessment. Twelve patients who were not currently experiencing hallucinations in any modality (as defined by a score of 1 or below in SAPS hallucination global score) were allocated to the NAVH group. The NAVH group consisted of four patients who had never experienced hallucinations, as well as eight patients with a history of hallucinations but currently not experiencing them as measured by the SAPS, as we aimed to study the impact hallucinations exert on the experimental task as a state, not as a trait. Patients reporting hallucinations (scoring at least 3 on the SAPS hallucinations global score) were allocated to the AVH group. None of the patients scored between 1 and 3 in this scale. A cut-off score was chosen based on previous studies (Allen et al., 2004; Shea et al., 2007). The AVH group subsequently completed the auditory hallucination subscale corresponding to the PSYRATS (Haddock et al., 1999). Finally, all subjects were assessed with the National Adult Reading Test (NART) (Nelson and Willison, 1991) to estimate premorbid intellectual performance (IQ) with a high test–retest reliability in schizophrenia (Morrison et al., 2000) (see Table 2).

**Table 2:** **Clinical, neuropsychological and demographic characteristics of the three groups**.

**Measures**	**Non-AVH mean (*SD*)**	**AVH mean (*SD*)**	**Controls mean (*SD*)**
N	12 (2 women)	15 (4 women)	16 (5 women)
Premorbid verbal IQ	110.42 (1.74)	112.14 (1.79)	112.69 (1.24)
Handedness scale	77.50 (16.43)	67.33 (15.96)	93.13 (5.06)
Age	37.83 (2.87)	41.73 (2.62)	42.69 (3.09)
Highest qualification achieved	2.25 (0.37)	2.07 (0.28)	2.44 (0.33)
Duration of illness (years)	15.17 (2.01)	15.33 (2.22)	
SANS total	10.88 (1.51)	10.40 (0.84)	
Affective flattening	1.71 (0.40)	1.53 (0.25)	
Alogia	1.79 (0.47)	1.43 (0.27)	
Avolition	2.29 (0.44)	2.27 (0.27)	
Anhedonia	2.17 (0.42)	2.57 (0.25)	
Attention	2.92 (0.43)	2.60 (0.30)	
SAPS total	4.92 (0.87)	9.90 (0.69)^**^	
Hallucinations	0.96 (0.23)	3.83 (0.24)^**^	
Delusions	1.17 (0.31)	2.97 (0.33)^*^	
Bizarre behavior	1.42 (0.31)	1.47 (0.25)	
Positive formal thought	1.38 (0.33)	1.63 (0.30)	
PSYRATS (hallucination subscale)	–	25.87 (1.73)	

SANS and SAPS: Scale for the Assessment of Negative and Positive Symptoms, respectively. PSYRATS: Scales to measure dimensions of hallucinations and delusions: the psychotic symptom rating scales.

Highest qualification achieved: 1 = compulsory education, 2 = sixth form education, 3 = higher education, 4 = postgraduate education.

** p < 0.001;

* p < 0.05.

There were no between-group differences in age [*F*_(2, 42)_ = 0.72, ns], education as measured by highest qualification achieved [1 = primary school, 2 = secondary school, 3 = 0 levels, 4 = A levels, 5 = university degree; *F*_(2, 42)_ = 0.35, ns], premorbid verbal IQ [*F*_(2, 42)_= 0.51, ns] and handedness measured by the Edinburgh Handedness Questionnaire [*F*_(2, 42)_ = 1.10, ns]. AVH patients had significantly higher total scores (*U* = 21.00, *Z* = −3.37, *p* < 0.05), hallucinations global subscale scores (*U* = 1.50, *Z* = −4.37, *p* < 0.001), and delusions global subscale *scores* (*U* = 26.50, *Z* = −3.18, *p* < 0.05) of the SAPS than NAVH patients. None of the other psychopathology measures revealed significant differences between patient groups (all *U* < 70.50, ns).

### Materials and procedure

#### Stimuli

The stimuli used in the experimental task were taken from the “Montreal Affective Voices” dataset (Belin et al., 2008). We used 30 non-verbal affect bursts of non-linguistic vocal sounds corresponding to emotions of anger, happiness and neutral expression recorded by 10 actors (five males). Since interaural intensity and length differences are known confounds in dichotic listening tasks (Hugdahl et al., 2008), voice stimuli were edited to equalize them in duration (900 ms) and amplitude (80 DB) using Audacity software (http://audacity.sourceforge.net). Male and female speakers were equally distributed across conditions.

#### Procedure

Written informed consent was obtained from each participant. The study and procedures were approved by the regional ethics committee from the NHS and Durham University Ethics Advisory Committee. All participants received £30 for their participation. Hearing loss and acuity differences between ears were measured using monaural white-noise bursts (duration 1 s) presented via headphones with various sound-pressure levels (steps of 10 dB). More details about this test can be found in (Hirnstein et al., 2007). Importantly, all participants had normal hearing and none reported hallucinations during testing. A mixed factorial ANOVA was performed with ear acuity (left ear, right ear) as within- and Group as between-subjects factors. In agreement with published data (Kannan and Lipscomb, 1974) the right ear (*M* = 30 dB) was slightly but significantly more sensitive than the left ear (*M* = 35 dB) [*F*_(1, 39)_ = 3.65, *p* < 0.05]. The main effect of Group and the interaction between Ear acuity and Group was not significant (all *F* ≤ 2.75, ns).

A pair of male/female voice stimuli (one of which was always neutral) were simultaneously presented, one to each ear, resulting in five possible combinations: Left angry/Right neutral, Left neutral/Right angry, Left happy/Right neutral, Left neutral/Right happy, and Left neutral/Right neutral (this last combination will be referred to as “baseline”). Participants were instructed to selectively attend to the voice presented to either the left or right ear and to decide on the sex of the speaker in the attended ear using a response box. Half of the participants used the left response key to indicate a female voice and the right to indicate a male voice. The response keys were counterbalanced across participants. A total of 144 trials were presented to each participant excluding five practice trials at the beginning of the experiment. In one block (72 trials), participants focused on voices presented to their right ear; in another block (72 trials), they attended to voices presented to their left ear. Block order was counterbalanced across participants. Participants listened to the stimuli and responded during the inter-trial interval of 3000 ms. Accuracy and RTs were analyzed. For the RT analysis, incorrect trials and outliers were excluded.

Degrees of freedom were epsilon-corrected (Greenhouse–Geisser) when sphericity was violated (Mauchly). *Post hoc t*-tests were alpha-adjusted (Bonferroni) for multiple comparisons.

## Results

Several previous studies suggest that emotional valence does not affect the relation between attention and implicit EP processing (Grandjean et al., 2008). Angry and happy stimuli were therefore collapsed to increase the trials number per condition and to simplify the experimental design.

### Accuracy (%)

**Figure 1:**
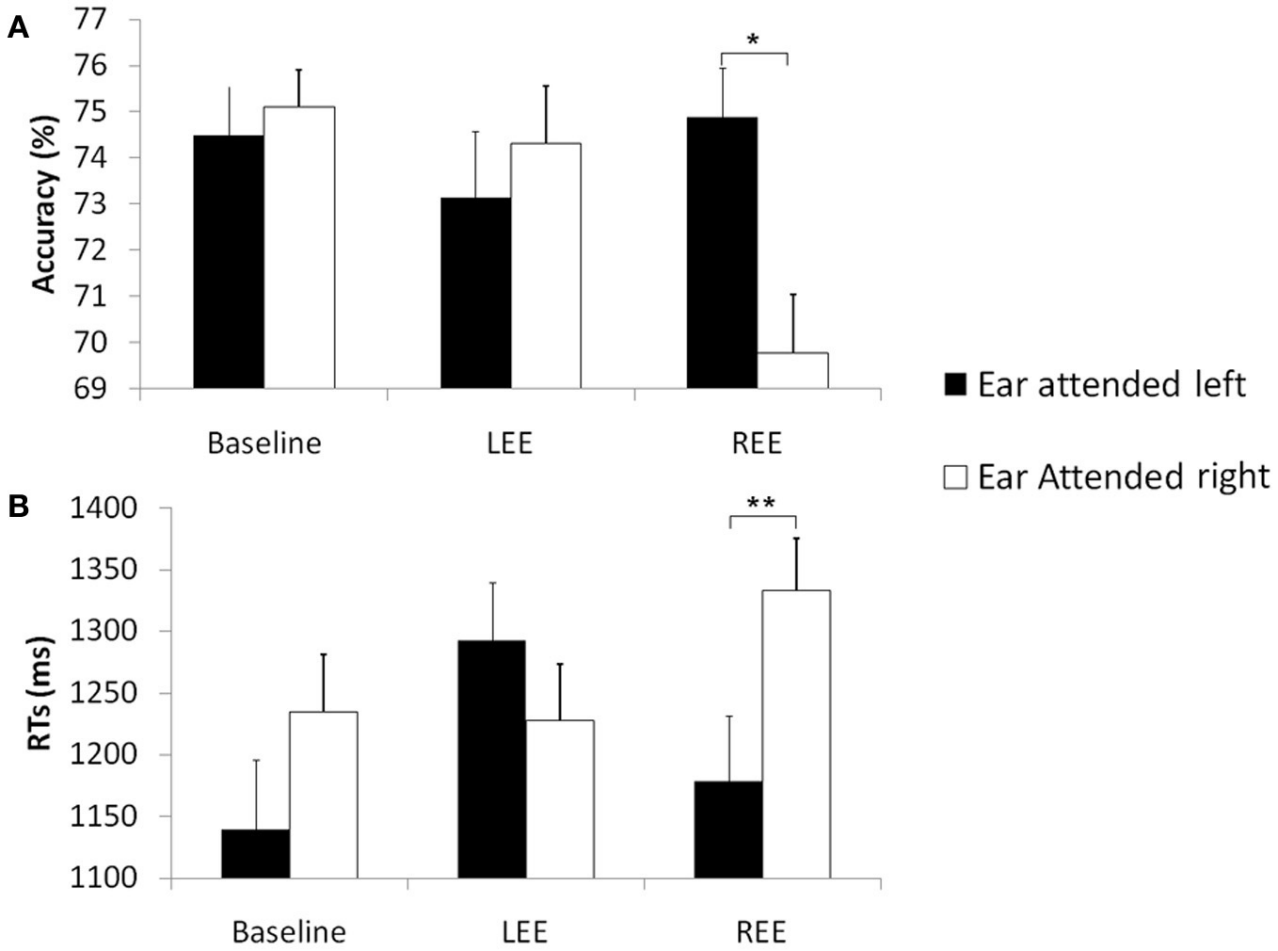
**Accuracy and RT in the gender prosody task across groups.** Mean accuracies (%) **(A)** and mean reaction times (ms), ^*^p < 0.05 **(B)** and standard errors in the sex discrimination task across groups [schizophrenia patients with hallucinations (AVH), without hallucinations (NAVH) and healthy controls] in three different conditions (baseline), trials with emotion presented to the left ear (LEE) and trials with emotions presented to the right ear (REE). Black columns represent trials in which the left ear was attended. White columns represent right ear attended trials, ^**^p < 0.001.

Participants' overall accuracy (across all groups and conditions) in the sex discrimination task was significantly above chance [*M* = 73% ± SD = 0.05, *t*_(40)_ = 20.1, *p* < 0.001, one-sample *t*-test, chance level was 50%]. Accuracy scores (%) were subjected to a 2 × 3 × 3 mixed ANOVA with Ear attended (left, right) and Trial-type [neutral binaurally (baseline), emotion on the left ear (LEE), emotion on the right ear (REE)] as within-participants factors and Group (AVH, NAVH, HC) as between-participants factor. The ANOVA revealed a main effect of Trial type [*F*_(2, 76)_ = 3.34, *p* < 0.05]. Alpha adjusted pairwise comparisons indicate that the accuracy during baseline (75.00% ± 0.01) is higher than during the REE condition (72.00% ± 0.01, *p* < 0.05). The LEE condition (73.40% ± 0.01) did not differ from REE and baseline. The main effect of Ear attended did not approach significance [*F*_(1, 38)_ = 0.42, ns]. Moreover, there was a significant interaction between Ear attended and Trial type [*F*_(2, 76)_ = 7.94, *p* < 0.05]. Alpha-adjusted *post hoc t*-tests revealed that in the REE condition, participants obtained lower accuracies when the right ear was attended (69.40% ± 1.27) than when the left ear was attended [75.00% ± 1.07, *t*_(41)_ = −3.09, *p* < 0.05], suggesting that if the emotion is presented to the attended ear, there is an increased difficulty to ignore the affective distractor while performing the sex discrimination task (see Figure 1A). No other *post hoc* test approached significance (all *t* ≤ 2.45, ns). No other main effect or interaction approached significance, (all *F* ≤ 1.49, ns).

### Reaction times (RTs)

Identical to the accuracy data analysis, a 2 × 3 × 3 ANOVA was calculated for RTs. As predicted, we found a significant main effect of Ear attended [*F*_(1, 38)_ = 9.33, *p* < 0.05] with significantly longer RT when attending to the right (1269 ms ± 39) than the left (1205 ms ± 43) ear, suggesting that, across the whole sample, sex discrimination by voice depends more the right hemisphere. Moreover, in agreement to our hypothesis that emotionally neutral trials are more efficiently processed than emotional trials there was significant main effect of Trial type [*F*_(2, 76)_ = 9.23, *p* < 0.001]; alpha-adjusted pairwise comparisons showed lower RT in the baseline (1187 ms ± 42) than in the LEE (1260 ms ± 38; *p* < 0.05) or REE (1256 ms ± 40; *p* < 0.05) conditions, again suggesting that, across the whole sample, EP interferes with the gender prosody discrimination task. The LEE condition did not differ from REE. Additionally, the main effect of Group was significant [*F*_(1, 38)_ = 5.45, *p* < 0.05]; alpha-adjusted pairwise comparisons revealed only lower RTs for AVH (1354 ms ± 65) than for HC (1062 ms ± 65, *p* < 0.05). Other group differences did not approach significance.

We predicted interference, if both processes that are gender discrimination (explicit task) and EP (implicit) information converge on the same side. The interference between both processes should be even stronger after stimulus presentation to the right ear, corresponding to the left hemisphere (inferior for prosody processing), as the non-specialized hemisphere cannot efficiently deal with both processes simultaneously. Accordingly, there was a significant interaction between Ear attended and Trial type [*F*_(2, 76)_ = 8.44, *p* < 0.001]; alpha-adjusted *post hoc t*-tests revealed a significantly slower RT for the REE condition when the right ear was attended [*t*_(40)_ = 4.35, *p* < 0.001], whereas LEE did not differ from the baseline condition (see Figure 1B), suggesting that the left hemisphere (inferior for prosody processing) has difficulties to perform the explicit task when the emotional distractor is simultaneously processed by the same (left) hemisphere.

**Figure 2:**
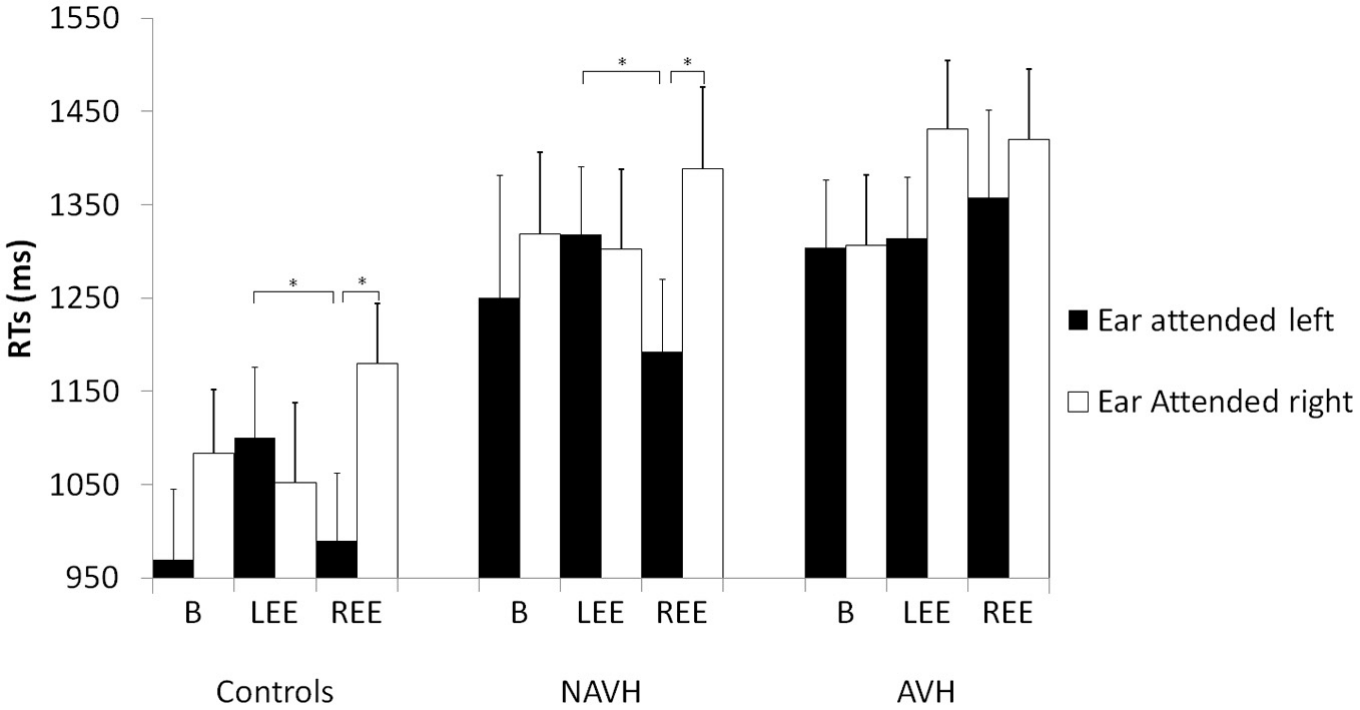
**RT in the gender prosody task across groups with three different stimuli pairs and two instructions (attending to either the right or left ear).** Mean reaction times (ms) and standard errors (*SE*) in milliseconds in the sex discrimination tasks for the different groups for the different groups [schizophrenia patients with hallucinations (AVH), without hallucinations (NAVH) and healthy controls] in three different conditions (baseline), trials with emotion presented to the left ear (LEE) and trials with emotions presented to the right ear (REE). Black columns represent trials in which the left ear was attended. White columns represent right ear attended trials. *Post hoc* tests are alpha-adjusted for multiple comparisons, ^*^p < 0.05.

Finally, there was a significant three-way interaction [*F*_(4, 76)_ = 6.74, *p* < 0.001]. This interaction comprised the factors Ear attended, Trial type and Group. To investigate this effect, three separate ANOVAs (one for each group) were performed. There was no main effect of Ear attended in any of the groups (all *F* ≤ 3.82, ns). The main effect of Trial type was only significant in HC [*F*_(2, 28)_ = 5.50, *p* < 0.05], and in the AVH group [*F*_(2, 28)_ = 8.74, *p* < 0.001]. In the control group, alpha-adjusted *post hoc* test showed faster responses in the baseline (1026 ms ± 68) than in the REE condition [1085 ms ± 68, *t*_(14)_ = 3.09, *p* < 0.05] but the baseline condition did not differ from LEE [1076 ms ± 68, *t*_(14)_ = 1.36, ns]. The REE and LEE conditions did also not differ significantly (see Figure 2). In the AVH group, the baseline condition (1309 ms ± 73) revealed faster RTs than REE [1395 ms ± 71, *t*_(14)_ = 3.01, *p* < 0.05] and LEE [1384 ms ± 77, *t*_(14)_ = 3.52, *p* < 0.05]. REE did not differ from LEE [*t*_(14)_ = 1.62, ns]. The main effect of Trial type in the NAVH group was not significant [*F*_(2, 20)_ = 0.37, ns]. Most importantly, the interaction between Ear attended and Trial type was significant in HC [*F*_(2, 28)_ = 28.07, *p* < 0.001, η^2^ = 0.513] and NAVH [*F*_(2, 20)_ = 15.45, *p* < 0.001, η^2^ = 0.480] but not in the AVH group [*F*_(2, 28)_ = 2.98, ns, η^2^ = 0.131]. The missing interaction between Ear attended and Trial type in AVH patients indicates that automatic processing of EP interfered with performing the explicit task in this group, regardless which ear was stimulated, probably as a result of aberrant lateralization in processing prosodic information.

To explain the significant interaction between Ear attended and Trial type in HC and NAVH, alpha-adjusted *post hoc t*-tests showed that RTs were slower when the Ear attended and the emotion coincided on the right [healthy controls: *t*_(14)_ = 3.70 *p* < 0.05; NAVH: *t*_(10)_ = 3.34, *p* < 0.05], not when the left ear was attended in the REE condition (all *t* ≤ 2.55, ns). The comparison between attended ears was not significant in LEE and the baseline condition (all *t* ≤ 0.85, ns). To compare convergent (Ear attended and side of emotion co-occurring at the same hemispace) and divergent condition (Ear attended and the side of emotion occurring at opposite hemispace), additional alpha-adjusted *post hoc t*-tests were performed. The analyses revealed that both control groups showed a significant difference between LEE and REE, when the left ear was attended [healthy controls: *t*_(14)_ = 4.88, *p* < 0.001; NAVH: *t*_(11)_ = −2.72, *p* < 0.05], not when the right ear was attended.

## Discussion

We investigated how involuntary attention is modulated by EP in AVH schizophrenia patients. By including a healthy control group and a NAVH patients group, general confounding effects of schizophrenia are controlled for, suggesting that abnormal modulation of involuntary attention by EP is associated specifically with AVH. Specifically, the findings show a right hemispheric bias for EP processing in NAVH and HC. AVH patients seem to be less lateralized for EP and therefore might be more susceptible to the interference caused by implicit EP processing; regardless which ear/hemisphere receives the emotional distractor. Such mechanism could explain why hallucinators focus on irrelevant features of the auditory environment and their attention is captured by the “voices.”

### Interference between implicit EP and explicit male/female voice discrimination

All participants had faster responses in the sex discrimination task when attending to the left (as opposed to the right) ear, in agreement with previous reports (Lattner et al., 2005; Sokhi et al., 2005). Likewise, all participants had faster responses on the emotionally neutral baseline condition than in emotional trials, also in agreement to previous findings (Aue et al., 2011) suggesting that interference with this task by auditory emotional stimuli is due to involuntary capture of attention. Also consistently with others (Grandjean et al., 2008), such interference was independent from emotional valence stimuli. The right hemisphere superiority for sex discrimination (Lattner et al., 2005; Sokhi et al., 2005) and EP (Ross and Mesulam, 1979; Ross et al., 1997; Friederici and Alter, 2004; Ross, 2010) may explain the longer RTs when the right ear was attended to, corresponding to the inferior-for-the-task left hemisphere, as an interhemispheric transfer is required.

We also found lower accuracies and slower responses when emotion was presented to the attended right ear, possibly due to interference caused by the convergence of explicit task and the (implicit) emotion presented in the same hemispace; perhaps because one cerebral hemisphere does not have the capacity to process both tasks in parallel. Interference was less pronounced when the explicit and implicit tasks occurred in opposite sides. Thus, it may be more difficult to allocate voluntary attention when the emotional distractor drives involuntary attention toward irrelevant but salient features of the target stimuli (Grandjean et al., 2005; Sander et al., 2005). Orienting attention away from the task-relevant hemispace probably makes emotional content of the prosody stimulus and its automatic processing less salient, consequently causing less interference.

The convergence of the emotional distractor and the attentional focus on the same side disrupted the performance significantly only when it happened on the right ear side, suggesting that the (inferior-for-the-task) left hemisphere is particularly sensitive to this effect. Sander and colleagues (Sander et al., 2005) observed the same convergence effect, but only in the right hemisphere; however, their sample was much smaller (15 subjects) and did not include patients with schizophrenia, perhaps reducing their ability to identify subtle behavioral change. Indeed, they did not find a significant behavioral asymmetry in the sex-discrimination task (Sander et al., 2005).

#### Interference differences in AVH patients

Our main finding is that AVH patients are most sensitive to interference effects from EP. Control and NAVH participants' performance decreased when the emotional voice was presented to the attended right ear. By contrast, in AVH patients EP interfered with performance regardless of the ear the distractor was presented to, or of the ear attended, suggesting that (1) AVH patients had difficulties controlling their selective attention during the presence of an emotional distractor and (2) AVH patients are not typically right lateralized for EP.

Firstly, although all groups are affected by emotional salience, both controls and NAVH overcome such difficulty when the salient stimuli are divergent to the ear attended, particularly if the emotional distractor does not converge with the superior-for-the-task attended ear. Conversely, AVH patients cannot benefit from the divergence effect. By contrast we did not find a general top-down deficit in AVH in comparison to NAVH patients, as both groups had comparable overall performance. On the other hand, our finding is consistent with reports suggesting that voluntary attention in schizophrenia may fail in the presence of salient bottom-up competitors (Hahn et al., 2010). Indeed, since AVH patients had shorter RTs at baseline than in the emotion conditions, even though they were generally slower in the sex discrimination task (including baseline trials), they particularly struggled when EP stimuli were present. Emotional salience of prosodic stimuli captures attention of AVH patients, regardless whether the interfering stimulus is in the focus of attention.

### Abnormal lateralization of emotional prosody in the AVH group

It still remains open whether this abnormal lateralization is a trait or a state in hallucinators. A previous study assessed phonological processing in two groups of schizophrenia patients, one with on-going AVH and another with history of AVH as well as healthy controls (Løberg et al., 2004). They implemented a dichotic listening paradigm in which participants either focused attention to the left or right ear, or performed a non-forced condition without specific attentional instruction (Løberg et al., 2004). They found that since abnormal dichotic listening asymmetry for language stimuli is a state marker for AVH which is coincident with the perception of AH, the modulation of dichotic listening performance by means of voluntary attention is a trait marker, seen both in patients with ongoing and with only a history of AVH (Løberg et al., 2004). This voluntary attention difficulty in dichotic listening might also relate to schizophrenia in general instead of a AVH trait in particular, however, our data shows that NAVH patients do not present such difficulty suggesting this deficit is an AVH trait.

Importantly, there is vast literature applying lexico-phonological dichotic listening tasks in schizophrenia patients (Green et al., 1994; Bruder et al., 1999; Levitan et al., 1999; Hugdahl et al., 2007; Force et al., 2008). Summarizing the cited studies, we can conclude that schizophrenia patients do not show the normal right ear advantage for lexico-phonological stimulus due to electrophysiological abnormalities in the left temporal lobe (Bruder et al., 1999) and reduced event related potential component of early auditory processing (Force et al., 2008). In relation to hallucinations, AVH patients show lack of ear advantage for linguistic stimulus in comparison to NAVH patient and controls (Green et al., 1994; Hugdahl et al., 2007; Ocklenburg et al., 2013), which are highlighted by gray matter reduction in the left temporal lobe (Hugdahl et al., 2007). Our study complements the literature by suggesting the existence of right hemisphere abnormalities in AVH patients reflected in abnormal lateralization of EP. It has previously been proposed that attentional control on dichotic listening reflects an interaction between frontal executive processing and the left and right temporal lobe speech processing, underlied by frontotemporal connections (Løberg et al., 2006; Hugdahl, 2009), which seems disrupted in AVH patients (Lawrie et al., 2002).

AVH patients could be more symptomatic and thence more cognitively impaired than NAVH patients, but in our sample there were no differences in premorbid verbal IQ. Unfortunately, we cannot fully rule out that a greater general cognitive impairment in the AVH group might have caused top-down difficulties in hallucinators. However, it should be noted that the NAVH and AVH groups did not differ in the baseline condition. Moreover, it is negative, rather than positive, symptoms that are associated with neurocognitive impairment (Lewandowski et al., 2007; Ventura et al., 2009). AVH patients rated higher on the delusion subscale, and therefore we cannot exclude delusions contributing toward the interference of implicit EP. However, impaired performance in dichotic listening in psychotic patients relates to hallucinations particularly (Løberg et al., 2002). We found no differences in hearing thresholds, thus a hearing deficit cannot explain the observed effects. Importantly, we do not disregard the impact that audio perceptual abnormalities such as pitch perception can exert on prosodic processing, which was demonstrated in previous research (Leitman et al., 2005, 2007, 2011). We did not test pitch perception specifically, as it would have exceeded the scope of the study. Both strand of research (pitch perception abnormalities and EP deficits in AVH) do not invalidate each other, but instead they are regarded as complementary. Importantly, in a previous study (Alba-Ferrara et al., 2011) we demonstrated that the brain representation of emotional processing reminds unchanged when statistically controlled for pitch differences.

AVH patients are not typically right-lateralized for EP because they do not benefit from attending to either ear during baseline, and convergent emotion does not deteriorate performance preferentially on either side. In addition, AVH patients' inability to filter emotion trials when the attended ear diverges from the ear in which EP is presented may support lack of hemispheric specialization for implicit EP processing. Although our study included two potentially right lateralized processes (EP and sex discrimination), we believe that the atypical lateralization rather refers to EP because of the significant main effect of ear attended. The left ear advantage for the explicit task was present for all groups. Atypical asymmetries in EP processing in AVH patients might not only help to understand the formation of hallucination and its cognitive impairments. They might also be useful as a diagnostic marker and risk factor for AVH formation in schizophrenic patients.

### Limitations

Several caveats are needed. Since all our patients took antipsychotic medications, we cannot fully ascertain whether the differences between groups are not influenced by them. The ability to ignore irrelevant stimuli, which is compromised in schizophrenia (Leumann et al., 2002), improves with atypical antipsychotics (Green et al., 2001). The three groups did not differ in premorbid IQ, education, years of illness, age, or handedness; thus, these factors are unlikely to explain the findings. Lastly, a mood disorder screening was performed ensuring that our patients were free from such confound. It should, however, be noted that patients had in general longer latencies compared to controls. Slow RT in general have been long recognized as one of the behavioral deficits of schizophrenia (Cromwell and Held, 1969). However, since the NAVH and the control group show the same pattern performance (accuracy and RT across conditions), the AVH group behaves differently in that they do not show the convergence-dependent interference effect.

### Clinical and theoretical implications

The present findings have implications for neuropsychological models of hallucinations. According to Woodruff (Woodruff, 2004), an attentional bias toward emotional stimuli in schizophrenia patients has been previously observed (Green et al., 2001; Waters et al., 2006) and it may underlie AVH. The present results extend the finding of abnormal attentional bias toward prosodic emotional stimuli presented to the non-dominant side regardless of the focus of voluntary attention in AVH patients specifically. Emotional salience of prosodic stimuli may capture attention even when it should be oriented away from the distractor, resulting in emotional inputs accessing processing in detriment of non-emotional competitors. Such attentional bias toward irrelevant EP stimuli might impair the ability to focus on relevant aspects of the acoustical environment (Javitt, 2009). A breakdown in selective attention may cause an overwhelming sensorial influx of irrelevant data resulting in abnormal perceptions (Alba-Ferrara et al., 2012). Hallucinations are aberrant perceptual processes which usually convey emotional salience. Such bottom-up saliency may diminish top-down control as AVH may shift attention toward the perceived voices (Hugdahl et al., 2007).

## Conclusion

The present study provides evidence that schizophrenia patients can implement top-down control as well as controls. Further, NAVH patients are as good as controls at controlling the effect of bottom-up salience (as manipulated by emotional distractors) on top-down control. Indeed, lower performance can be overcome if emotional distractors are presented to the non-attended ear and to the inferior-for-the-task hemisphere. AVH patients, however, cannot overcome divergent emotional distractors presented to the putative inferior-for-the-task side, suggesting that bottom-up salience may capture attention revealing inefficient top-down control. In AVH patients, a failure of the typical lateralization for EP as the core mechanism underlying abnormal modulation of attention by EP is suggested.

### Conflict of interest statement

The authors declare that the research was conducted in the absence of any commercial or financial relationships that could be construed as a potential conflict of interest.
